# Lineage Range Estimation Method Reveals Fine-Scale Endemism Linked to Pleistocene Stability in Australian Rainforest Herpetofauna

**DOI:** 10.1371/journal.pone.0126274

**Published:** 2015-05-28

**Authors:** Dan F. Rosauer, Renee A. Catullo, Jeremy VanDerWal, Adnan Moussalli, Craig Moritz

**Affiliations:** 1 Research School of Biology & Centre for Biodiversity Analysis, Australian National University, Canberra, Australian Capital Territory, Australia; 2 CSIRO Land and Water Flagship, Canberra, Australian Capital Territory, Australia; 3 Centre for Tropical Biodiversity & Climate Change, College of Marine and Environmental Sciences, James Cook University, Townsville, Queensland, Australia; 4 eResearch Centre, Division of Research and Innovation, James Cook University, Townsville, Queensland, Australia; 5 Sciences Department, Museum Victoria, Melbourne, Victoria, Australia; University of California Berkeley, UNITED STATES

## Abstract

Areas of suitable habitat for species and communities have arisen, shifted, and disappeared with Pleistocene climate cycles, and through this shifting landscape, current biodiversity has found paths to the present. Evolutionary refugia, areas of relative habitat stability in this shifting landscape, support persistence of lineages through time, and are thus crucial to the accumulation and maintenance of biodiversity. Areas of endemism are indicative of refugial areas where diversity has persisted, and endemism of intraspecific lineages in particular is strongly associated with late-Pleistocene habitat stability. However, it remains a challenge to consistently estimate the geographic ranges of intraspecific lineages and thus infer phylogeographic endemism, because spatial sampling for genetic analyses is typically sparse relative to species records. We present a novel technique to model the geographic distribution of intraspecific lineages, which is informed by the ecological niche of a species and known locations of its constituent lineages. Our approach allows for the effects of isolation by unsuitable habitat, and captures uncertainty in the extent of lineage ranges. Applying this method to the arc of rainforest areas spanning 3500 km in eastern Australia, we estimated lineage endemism for 53 species of rainforest dependent herpetofauna with available phylogeographic data. We related endemism to the stability of rainforest habitat over the past 120,000 years and identified distinct concentrations of lineage endemism that can be considered putative refugia. These areas of lineage endemism are strongly related to historical stability of rainforest habitat, after controlling for the effects of current environment. In fact, a dynamic stability model that allows movement to track suitable habitat over time was the most important factor in explaining current patterns of endemism. The techniques presented here provide an objective, practical method for estimating geographic ranges below the species level, and including them in spatial analyses of biodiversity.

## Introduction

The spatial distribution of diversity, both between and within species, results from a complex interplay of past and current processes, often strongly influenced by repeated climate cycling associated with glaciation, aridification and sea level change during the late Pleistocene [1,2]. Two factors are central to the distribution of intra- and inter-specific lineages: the ability of populations to persist in or move to areas of suitable habitat in the face of changing climate, and their ability to maintain gene flow despite persistent or episodic biogeographic barriers [3]. These factors result in evolutionary refugia [4] where longer-term stability of local or regional environments has sustained species or populations that were unable to persist in surrounding areas, often leading to elevated endemism. These factors also lead to biogeographic breaks—areas where turnover of species and lineage range boundaries are concentrated. Where these patterns of endemism and turnover are shared across multiple taxa, we can start to build a general picture of the contribution of climatic and topographic heterogeneity to the distribution of diversity in the taxa of interest. Importantly, an understanding of the specific locations where diversity is concentrated now will also contribute to better decisions about conservation, both for individual taxa and for landscapes and their biota.

The importance of stable evolutionary refugia in maintaining species diversity through late Pleistocene climate change is well known. Regions and landscapes across which climatic conditions remained more suitable from the last glacial maximum (LGM) to the present tend to have higher richness [5] or endemism of species [6,7]. Because phylogeographic structure within widespread species is especially sensitive to spatial dynamics of populations through the last glacial cycle [8] we expect comparative patterns of phylogeographic lineage endemism to provide even higher correspondence to locations of evolutionary refugia. Accordingly, models of relative stability of habitats are often able to predict hotspots of phylogeographic endemism within species or assemblages [9–13].

Comparative phylogeography has shown considerable capacity to identify evolutionary refugia and contribute to our understanding of how the response of species and their constituent lineages to past climate fluctuation has shaped current diversity. However, it remains a challenge to consistently estimate the geographic ranges of intraspecific lineages and thus infer phylogeographic endemism across taxa. This is because sampling for genetic analyses is typically sparse relative to all species occurrence records. A range of techniques exist to model gradients of genetic or morphological variation (such as [14,15]). However, a method is needed that allows us to represent the distribution of discrete lineages, not the gradients of variation within them. The simple approach of estimating lineages boundaries as the midpoint between known locations of adjacent lineages [10,16] risks inferring an arbitrary hard boundary, typically in sampling gaps, which are the very locations of least certainty. Here we develop a probabilistic approach that integrates information from both sequenced locations and all species records, and demonstrate this for rainforest frogs and lizards from eastern Australia.

**Fig 1:**
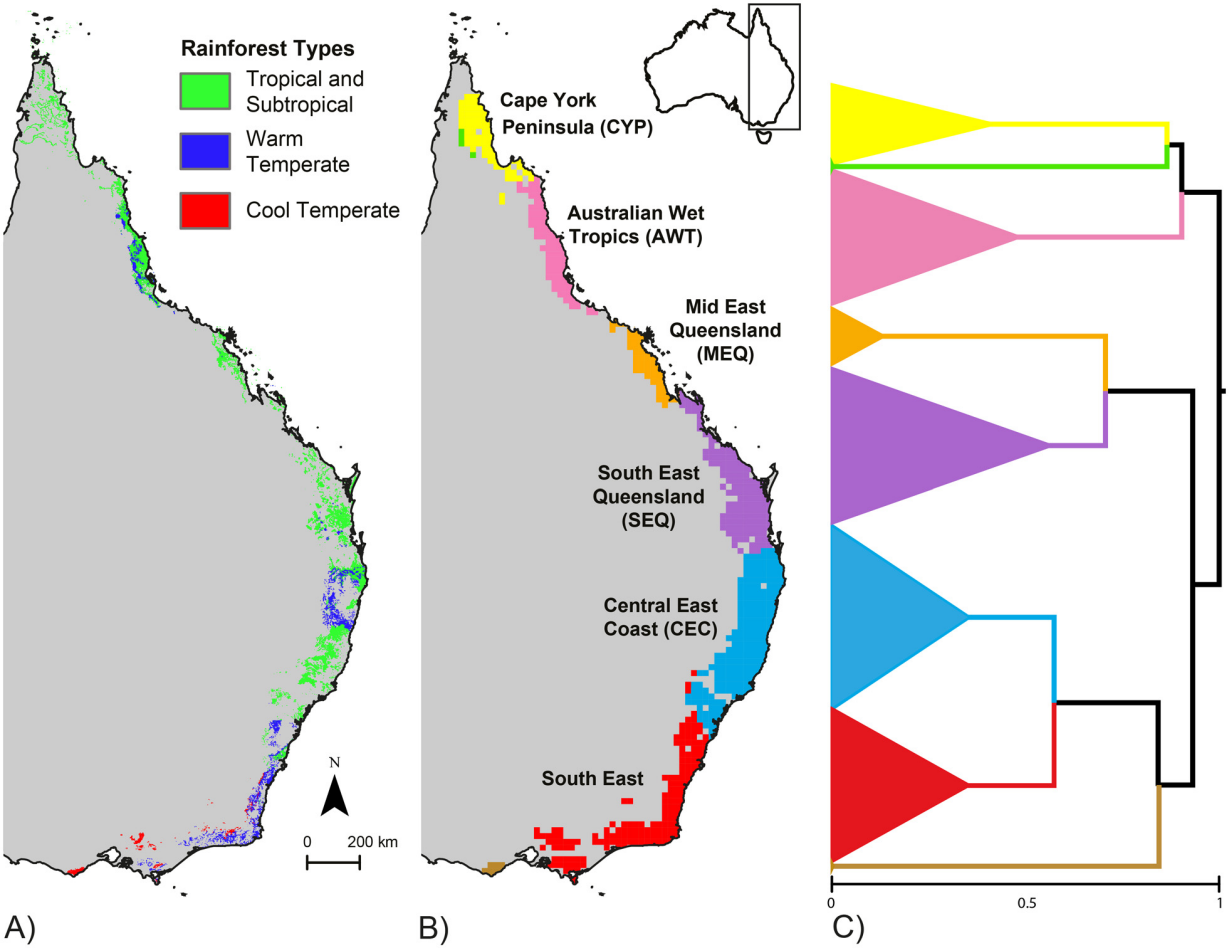
A) Distribution of rainforest types in mainland eastern Australia. B) Bioregions defined by similar lizard and frog lineage composition based on hierarchical clustering of the lineage distribution models. C) A dendrogram of the affinity between lineages showing deep splits between the AWT and MEQ regions, and a second major break at the Border Ranges, dividing the SEQ and MEQ regions to the north, from CEC and SE to the South. The horizontal axis represents Bray-Curtis dissimilarity of lineage composition from identical composition (left edge) to no shared lineages at the root.

The patchwork of rainforests that span Australia’s east coast, across 3500 km from Cape York to Tasmania (Fig 1a), provide an excellent opportunity to study diversification and persistence though shifts in the location and connectivity of suitable habitat. These naturally disjunct rainforest areas represent environmentally and compositionally distinct ‘islands’ of a cooler-wetter ‘mesotherm archipelago’, which are surrounded by warmer-drier sclerophyll woodlands [17]. These areas reflect a general contraction of Australian rainforests to the mesic east coast from the mid-late Miocene [18]. Whilst more dispersive taxa are able to maintain connectivity between patches [19,20], low-dispersal rainforest-specialist taxa show high levels of endemism and turnover across regions [21–24]. The legacy of the Plio-Pleistocene climatic oscillations is well documented for Australian Wet Tropics of north-east Queensland, where spatial variation in habitat stability since the LGM explains both phylogeographic diversity and species richness in low dispersal taxa [5,25,26]. However, there have been few studies relating spatial modelling of stability or otherwise inferred refugia to patterns of intra- and inter-specific diversity across the whole Eastern Australian rainforest system [19,21].

We focus here on low-dispersal rainforest endemic frogs and lizards, for which species distributions are well documented and there is reasonably extensive phylogeographic data. We develop and apply a method to model the distribution of evolutionarily distinct lineages within these species, and, combining data across all sampled taxa, identify areas of highest lineage endemism. We then ask four questions: 1) Which areas share similar composition, and where are the boundaries of such areas, thus reflecting patterns of turnover common to multiple taxa? 2) Where are the concentrations of endemic lineage diversity for rainforest lizards and frogs? 3) Where was the most stable habitat for rainforest through past climate changes, and 4) To what degree can this historical stability explain current patterns of endemism in Australia’s eastern rainforests?

## Methods

### Species and lineage distributions

We identified 52 lizard and 42 frog species that are principally restricted to Australian rainforest, and drawing on this list, compiled data from published and unpublished phylogeographic studies to identify evolutionarily distinct lineages within a large sample (final N = 53). Because both richness of rainforest-restricted species and our phylogeographic data attenuate rapidly in the cooler rainforests of the south, our analyses of lineage endemism exclude the south-eastern continental and Tasmanian rainforests.

Lineages were identified from the studies in Table 1, where a lineage represents a historically isolated, independently evolving population, equivalent to Evolutionarily Significant Units [27]. For our study, lineages were defined using the cited studies’ identification of strongly supported unique clades. Despite the variation in sequencing methodology and taxonomic attention across the literature, lineages were principally defined by deep mtDNA divergence, often with corresponding divergence at nuclear loci [25] and / or in phenotype [28,29]. Our principal interest for this study was in the distribution of evolutionary lineages, not their taxonomic status, so discrete, independently evolving evolutionary units were treated as lineages, whether recognized in current taxonomy or not. We obtained known locations for 102 identified lineages (based on sequenced specimens) within our 53 rainforest specialist species (Table 1). Lineages were identified from many separate phylogenies using different genes and varying methods, thus we do not scale measures of endemism by unique branch lengths as is done for phylogenetic endemism [30].

**Table 1:** Lineages of rainforest specialist lizards and frogs included in the study.

Family	Genus	Species	Lineages	# of lineage records	Data sources
**Agamidae**	*Hypsilurus*	1	2	73	[25]
**Carphodactylidae**	*Carphodactylus*	1	4	188	[31]
**Carphodactylidae**	*Phyllurus*	10	10	1259	[28,32,33]
**Carphodactylidae**	*Saltuarius*	5	6	339	[31,34,35]
**Gekkonidae**	*Cyrtodactylus*	5	5	71	[29]
**Hylidae**	*Litoria*	6	14	407	[36]
**Myobatrachidae**	*Mixophyes*	3	3	115	[37]
**Scincidae**	*Carlia*	2	5	1033	[33,38]
**Scincidae**	*Eulamprus*	3	6	1053	[25,33]
**Scincidae**	*Glaphyromorphus*	1	3	69	[25] & S1 Data
**Scincidae**	*Gnypetoscincus*	1	2	1351	[25,31]
**Scincidae**	*Lampropholis*	3	9	1084	[12] & S1 Data
**Scincidae**	*Saproscincus*	12	33	941	[39,40]
	**Total**	**53**	**102**	**8703**	

For each species we collated: (a) locations for sequenced specimens of each identified lineage based on published studies and our own and collaborators collections (Table 1 and S1 Data), and (b) known locations for each species, without genetic data to specify their lineage, from museums and other collections via the Atlas of Living Australia (ALA; www.ala.org.au). However, there are unsampled locations where the species could be expected to occur based on habitat suitability. In order to estimate the range and thus endemism of lineages, we needed to extrapolate to the overall lineage extent from known (sequenced) lineage locations.

To address this limitation, we developed a new approach to probabilistically estimate the likely distribution of intraspecific lineages. The goal is to estimate how likely it is that a given intraspecific lineage occurs in each grid cell. One approach to this problem is to assume that if a species is known to occur at a given location, but the intraspecific lineage at that location has not been determined, it is more likely to be a member of the closest known lineage, than of more distant lineages. A recent study addresses this problem [41] by fitting a model of semivariance to represent the likelihood of lineage similarity as a function of distance. However, suitability of the intervening habitat may facilitate or block movement between locations, and could be as important as geographic distance in determining the ability of a lineage to disperse to a given location. We thus modelled lineage occurrence as conditional on (i) the habitat suitability of the cell for the species as a whole; and (ii) the degree to which the cell is connected by suitable habitat to known locations of each lineage.

**Fig 2:**
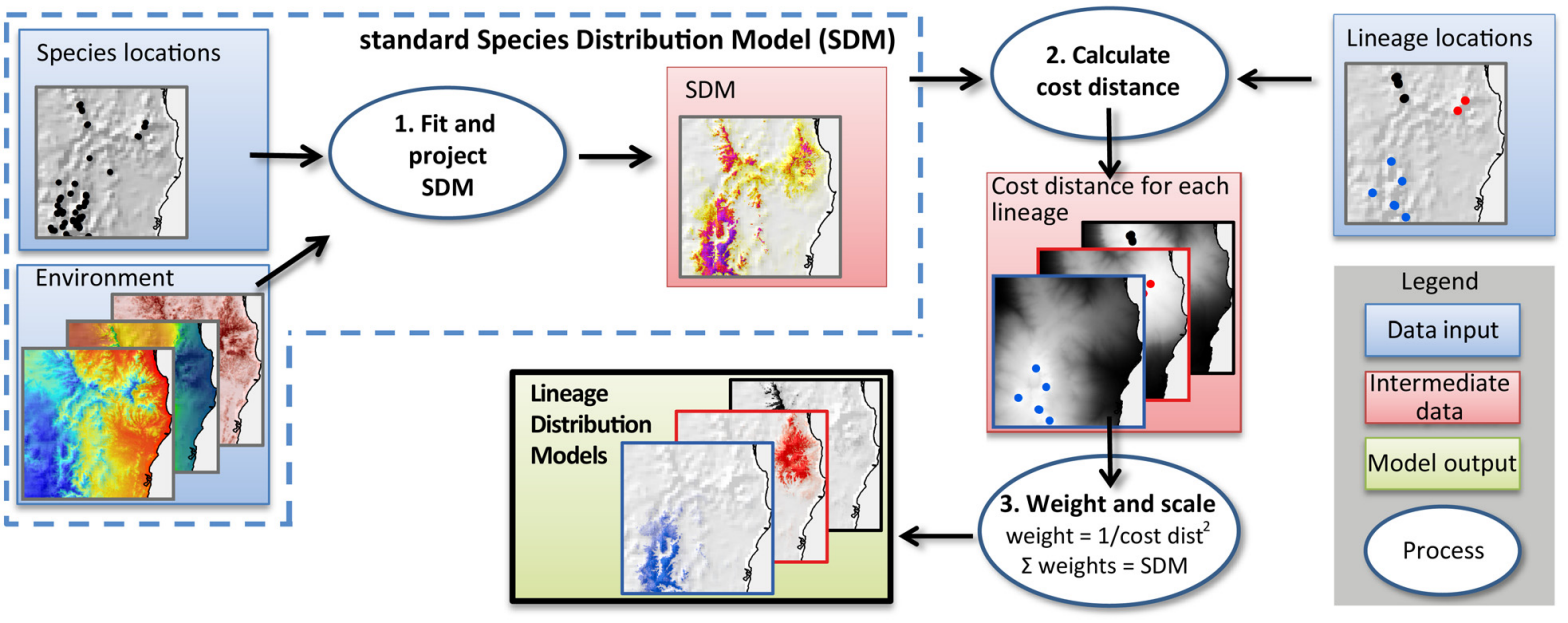
Steps in the lineage distribution model (LDM) method. First a species distribution model is fitted, using established methods (step 1). Then, a cost distance calculation is used to determine, for each pixel, its connection through suitable habitat to known locations of each lineage (step 2). Finally, the cost distance is turned into a weight, and scaled so that for each pixel, the weights for the lineages sum to the species model suitability (step 3). In effect, the species model is partitioned among the known lineages in proportion to their likely level of connectivity to that location.

To reflect these conditions in a spatial model (Fig 2), we first generated a species distribution model (SDM) for each species in MaxEnt version 3.3.3 [42] at 0.01 degree resolution using the first 19 Bioclim variables [43] to represent annual and seasonal temperature and precipitation. Slope and topographic wetness index (TWI; [44]) were calculated at 0.01 degree resolution and also used as model predictors. We restricted background points to a radius of 2.5 degrees (~275 km) around the location records for the species so that the models would emphasize those factors relevant locally in distinguishing suitable from unsuitable sites. Species location records were, in general, biased towards more accessible areas, as is typical for Australian datasets. We followed *Phillips et al*. [45] in dealing with spatially biased samples, such that background points for each species were selected at random from sites where any member of that species’ family had been recorded. Default values were used for other MaxEnt settings, including default prevalence of 0.5 and regularization multiplier of 1. We checked models against published species ranges and removed areas of over-prediction beyond the species’ extent of occurrence.

We then partitioned the modelled habitat suitability value for the species, dividing the SDM suitability value for each cell among the intraspecific lineages in proportion to the square of the distance to the nearest known location of the lineage. To take account of barriers to dispersal, we measured cost distance rather than linear distance to the nearest known location of each lineage. The cost of traversing each cell, for the cost-distance analysis, was calculated from the SDM for the current species, as -log(habitat suitability) [5]. The lineage models were run using Python 2.7 scripts to automate functions in ArcGIS [46] and MaxEnt [42]. Scripts are available from github.com/DanRosauer/phylospatial.

**Fig 3:**
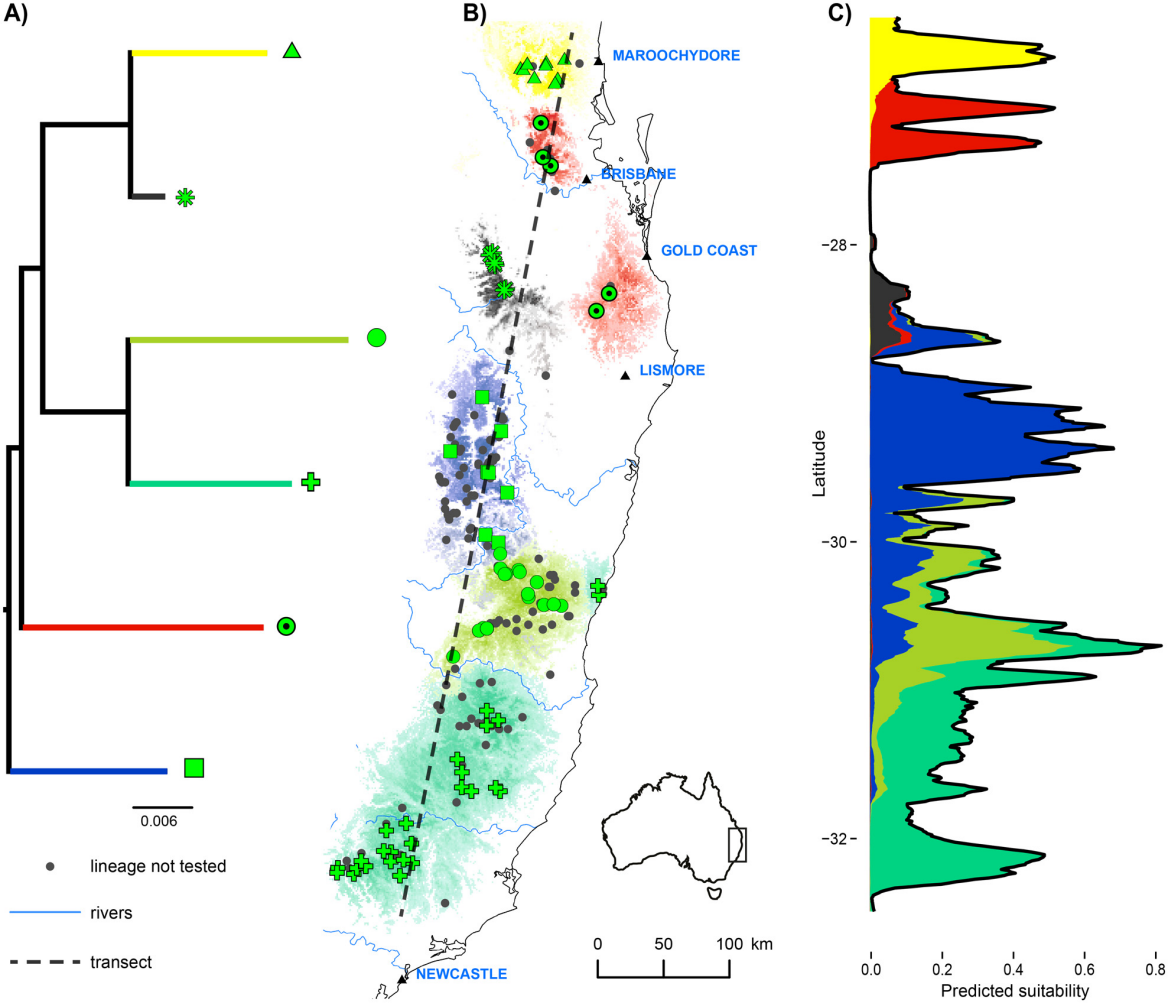
Example of lineage distribution models for 6 lineages of *Saproscincus rosei*. A) Phylogenetic relationships between the lineages. B) Known locations of each lineage (green symbols) are supplemented by locations of specimens (black dots) where the presence of the species is known, but not the lineage identity. For each lineage, the relative likelihood of occurrence is shown with colors matching the corresponding tree branch. C) The likelihood of occurrence of each lineage along the dashed transect in B is shown in the same colors. In this stacked plot, the total value (black line) is the predicted suitability for the species, partitioned between the lineages. Values for several lineages at the same location represent uncertainty of lineage occupation, not co-occurrence.

The resulting 102 models estimate the likelihood of each lineage occurring at each cell, with the likelihood values across all lineages scaled to sum to the species-level SDM habitat suitability value for that cell. We thus avoid placing sharp boundaries on the distribution of lineages except where there is evidence for such boundaries, instead creating a gradient of likelihood informed by known locations for the lineages and species-level habitat suitability. This method is consistent with the observation that the transition or contact zone between lineages is more likely to occur in areas of lower habitat suitability [25]. An example of this approach for the 6 lineages identified in the skink species *Saproscincus rosei* ([47], Moussalli *et al*. unpublished) is shown in Fig 3, with the likelihood of occurrence for each lineage resulting from the interaction of habitat suitability for the species, proximity to known locations of each lineage, and the suitability of the intervening areas as habitat.

An alternative modelling approach would be to use a standard SDM technique to model the distribution of each intraspecific lineage separately, based on its realized environmental niche. However, this approach would treat the different distribution of each lineage as determined entirely by differences in its ecological tolerances, an assumption which is difficult to prove [48]. Such models could also be compromised by a small sample size per lineage. Our new approach assumes a common environmental envelope for each species, with lineage boundaries within the species informed by isolation of areas of suitable habitat, but not niche differentiation.

### Biogeographic regions

To assess compositional similarity and turnover from the lineage distributions (Aim 1), we summarized the lineage models to 0.2 degree grid cells, taking the sum of lineage distribution model (LDM) values within the cell for each lineage. We then calculated a dissimilarity matrix, comparing the lineage composition of all pairs of cells using the model likelihood for each lineage as the abundance term in the Bray-Curtis function, and performed a hierarchical cluster analysis to group compositionally similar areas. These steps were performed in Biodiverse version 0.19 [49].

### Estimating richness and endemism

We calculated species richness as the sum of the habitat suitability scores across all species models. Species richness is sometimes calculated by first thresholding models to translate suitability into predicted presence and absence, but this has been found to introduce systematic biases [50]. Instead, following the advice of Calabrese *et al*. [50], we summed the suitability scores directly [51], so a low suitability score for a grid cell makes a small contribution to richness in that cell. The resulting values should be interpreted as a probabilistic measure of richness, not as an absolute count of species. Because the lineages are parapatric components of species, their models sum to the species model (Figs 2 and 3c), and the value for lineage richness is thus the same as species richness, not a meaningful separate measure. We also calculated a variant of range-weighted endemism (WE; [52]) of the lineages (Aim 2). When applied to species, WE sums the proportion of the total geographic range of each species that occurs in each cell, as the sum of ^1^/_range size_ across all species. This is equivalent to counting species to measure richness, but with the value of each species divided equally across all of the cells where it occurs, so an area has high WE where it contains a large proportion of the range of a number of species.

To adapt the WE method to assess lineage endemism directly from modelled ranges of individual lineages, instead of summing the proportion of the total range of each lineage that is found in a cell, we instead summed the LDM value in a cell, as a proportion of the total LDM values for that lineage across all cells. This gives a likelihood-based assessment of endemism, which we call *model weighted endemism (MWE)*. Where *l* is one of *n* lineages, each with an LDM, we calculate MWE for each cell as follows:

$$MWE=\sum_{1..n}^{l}\frac{localsuitability_{l}}{totalsuitability_{l}}$$

Endemism can also be estimated from modelled ranges [16] by setting a threshold to turn the habitat suitability values into binary presence / absence, but as discussed above for species richness, we used the full range of model variation as a less biased estimate of occurrence, and to propagate the unthresholded values through all of the analyses flowing from the occurrence models. Very small LDM suitability values (<0.01) extended over wide areas, and were excluded to simplify subsequent calculations.

We use the above methods to calculate the difference between patterns of species and lineage endemism, which can highlight areas where widespread species contain localized variants. This may also indicate locations of refugial persistence that are masked at species level by subsequent range expansion.

### Estimating late Pleistocene climate stability

Paleo-climate habitat stability can be estimated by fitting an SDM to the current environment of the biome of interest, and projecting this model back into past environments to infer the degree of continuity of suitable habitat through a succession of time periods. Assessments of paleo-climate stability have typically analyzed changes in climate back to the LGM (21ka; [5,6,9,11,53]), but recently global circulation models (GCMs) have become available for a series of earlier time periods back to the last interglacial (LIG, 120 ka). The LIG was a period of globally higher temperatures, and these data have now enabled analysis of the role of longer-term climate stability in shaping current biodiversity [10,54]. We applied the stability analysis method and climate datasets following the method of Fuchs *et al*. [55], based on the Hadley Centre Climate model (HadCM3; [56]).

There is wide variation between the predictions of paleo-climate GCMs, and modelling with multiple GCMs might be preferred [57]. However, we chose to use the HadCM3 model alone as it shows far greater agreement with LGM paleo-climate proxies across the Asian-Australian tropics than do other available simulations [58]. The HadCM3 dataset consists of climate reconstructions for 62 time slices: from the present back to the LGM (22 ka) in 1000-year intervals, then 2000-year intervals until 80 ka, and then every 4000 years up to 120 ka. Downscaling of climate surfaces from the native resolution of 50 x 50 km was performed using the *climates* package [59] in R (www.r-project.com). For each time slice we generated eight of the widely used Bioclim variables [43]: mean annual temperature (Bioclim 1), temperature seasonality (Bioclim 4), mean temperature of the warmest and coldest quarters (Bioclim 10 & 11), mean annual precipitation (Bioclim 12), precipitation seasonality (Bioclim 15), precipitation of the wettest quarter (Bioclim 16), and precipitation of the driest quarter (Bioclim 17). For the correlation structure of the variables see S1 Table.

Topography has strong effects on the distribution of vegetation types, both as a driver of topo-scale climate regimes [60] not captured by GCMs or macro-climate interpolations [43], and also through its direct effects, such as on accumulation of soil and moisture and spread of fire. Estimating past habitat suitability using climate alone may thus exaggerate instability by emphasizing a highly variable component of habitat (climate), while omitting other more stable factors, such as topography. For our rainforest SDM we included two topographic predictors of habitat suitability (slope and elevation range) calculated from the Etopo1 digital elevation model [61] at 1 minute resolution (~1.8 km) and summarized (median slope, total elevation range) to align with the 2.5 minute climate grids. To include areas currently under sea (for past time periods), slope and elevation range were calculated from combined land and bathymetric elevation data.

To capture the full climatic range of rainforest, we derived the current extent all east Australian rainforest (including Tasmania, see S1 Fig) from the National Vegetation Information System (NVIS 4.1; [62]). We used modelled extent prior to European settlement (NVIS pre-1750 dataset) to better reflect the full environmental space suitable for rainforest [53]. All rainforest types (Fig 1a) were combined and resampled from the 100 m resolution to 2.5 minutes to match the paleo-climate surfaces. Pixels containing any rainforest were classed as rainforest to include areas where rainforest occurs principally in small patches. We modelled the environmental niche of the rainforest pixels using MaxEnt [42], with background environment drawn from 10000 random locations within 200 km of areas of pre-clearing rainforest. The model was then projected to each of the 62 time slices in the HadCM3 climate time series, to estimate the distribution of suitable rainforest habitat at each time, using clamping to limit prediction into climate space outside the range of conditions available to train the present day model. Topography was treated as constant over this time period.

We calculated static stability (Aim 3) as the sum of the negative log of suitability through time for each cell, and took the exponent of this value to give to a habitat stability value (ranging from 0 to 1), representing the degree to which that cell has continuously provided suitable climate for rainforest [5]. At the extremes, 1 indicates continuous suitability for rainforest within a grid cell over all time periods, while 0 indicates that the grid cell was entirely unsuitable for rainforest during all or some periods.

An area of suitable habitat that functions as a refuge over geological time need not be a static location, instead moving to track suitable conditions, for example by shifting downslope in cooler times. In the dynamic stability model we allowed continuity between suitable locations by specifying a maximum velocity of movement, to represent the ability of rainforest to expand into newly suitable areas nearby. This model, described in detail by Graham *et al*. [5], estimates suitability at each time period, and allows dispersal between time periods limited by a cost distance function—where cost is a function of habitat suitability—and by a maximum dispersal distance calculated for a particular maximum dispersal speed. We used a speed of 10m yr^-1^, the median of the range of estimated expansion speeds of rainforest, trialed in the original dynamic stability study [5]. Under dynamic stability, a grid cell is considered stable if rainforest was able to persist in the cell or to disperse to it from other areas occupied by rainforest.

### Relating stability and endemism

To assess the degree to which paleo-climate stability can explain lineage endemism (Aim 4), we fitted generalized linear models (GLM) for all rainforest pixels, with lineage endemism as the response, log transformed to meet model assumptions of normality. As well as static and dynamic stability, the models included current suitability for rainforest and a range of current environmental factors which could be expected to influence endemism: mean annual temperature, annual precipitation and precipitation of the driest quarter, and topographic roughness calculated as the standard deviation of elevation in each 2.5’ cell. The total area of rainforest available as habitat in each region could be a constraint on lineage range sizes and thus on endemism. To control for this, the total rainforest area in each region (eg 169 rainforest cells in MEQ) was assigned as a property of each cell in the region and included as a model covariate. The predictors were standardized to μ = 0, σ = 1. The set of supported models, limited to four environmental predictors per model plus region area, were combined using corrected Akaike Information Criterion (AICc) weight, determined in the *MuMIn* package in R. To test for the effect of local spatial autocorrelation, we reran the same model selection procedure using a spatial autoregressive error model (function errorsarlm in R package *spdep*; [63]) instead of GLM. To confirm the relative importance of the predictors in explaining lineage endemism, we also fitted boosted regression tree models of lineage endemism using the same predictors in R package *Dismo* [64,65].

## Results

### Compositional turnover

The regions identified by clustering of compositional similarity (Fig 1) show spatial coherence and clear boundaries, largely matching known biogeographic breaks [66]. The deepest division was at the southern boundary of the Australian Wet Tropics (AWT) region (the Burdekin Gap), where the AWT and Cape York Peninsula (CYP) were separated from everything to the south. The boundary between Mid East Queensland (MEQ) and South East Queensland (SEQ) occurred at the St Lawrence Gap, a dry barrier to rainforest species. A major division occurred near the Queensland—New South Wales border, separating SEQ from the Central East Coast (CEC) and areas further south, bisecting an apparently continuous and diverse area [67] known as the Macleay-MacPherson Overlap [68]. The southern boundary of the CEC region runs through the Sydney basin. Two sets of pixels, green and brown in (Fig 1B & 1C) came out as highly distinct clusters, most likely due to the small subset of rainforest species in our study occurring in these peripheral areas.

### Stability

**Fig 4:**
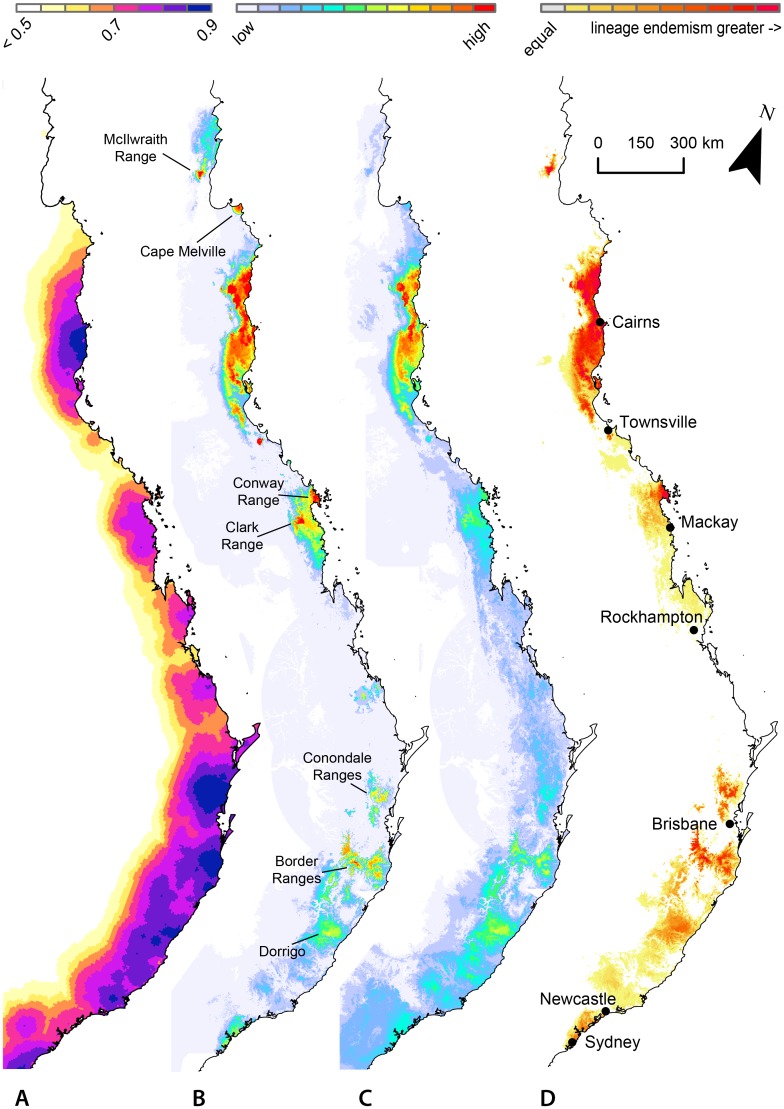
A) Paleo habitat stability of rainforest since 120 ka using the dynamic stability model allowing dispersal at 10 m yr^-1^. B) Model-weighted lineage endemism peaks in the AWT just north of Cairns, and shows distinct highlights in a small subset of each rainforest region, such as Cape Melville in CYP, Conway Range and Eungella in MEQ, and the Border Ranges just south of Brisbane. C) Model-weighted species richness. D) Lineage endemism minus species endemism. Red areas have far greater lineage than species endemism, identifying concentrations of spatially restricted diversity, which would be missed in a species-level analysis. Colors by quantile classes for each map. Note that for compact map presentation, north is offset by 20° as indicated.

The SDM for present rainforest distribution returned an AUC value of 0.86, with occurrence of rainforest most strongly related to annual precipitation, temperature seasonality and slope (S2 Table). Projecting this model into the past, we generated a stability surface based on the realized climate niche for all rainforest (Fig 4A and S1 Fig). The most stable areas in our dynamic stability analysis were the southern AWT, the Conondale Ranges area in SEQ and a series of discrete areas in the CEC region the largest of which is the Border Ranges. Stability in rainforest areas of the CYP region and northern AWT was very low. Modern day rainforest occurs across a wide range of historical stability, including low stability areas where its occurrence was likely less continuous through time, allowing comparison the effects on endemism of different histories of stability within current rainforest.

### Lineage endemism

**Fig 5:**
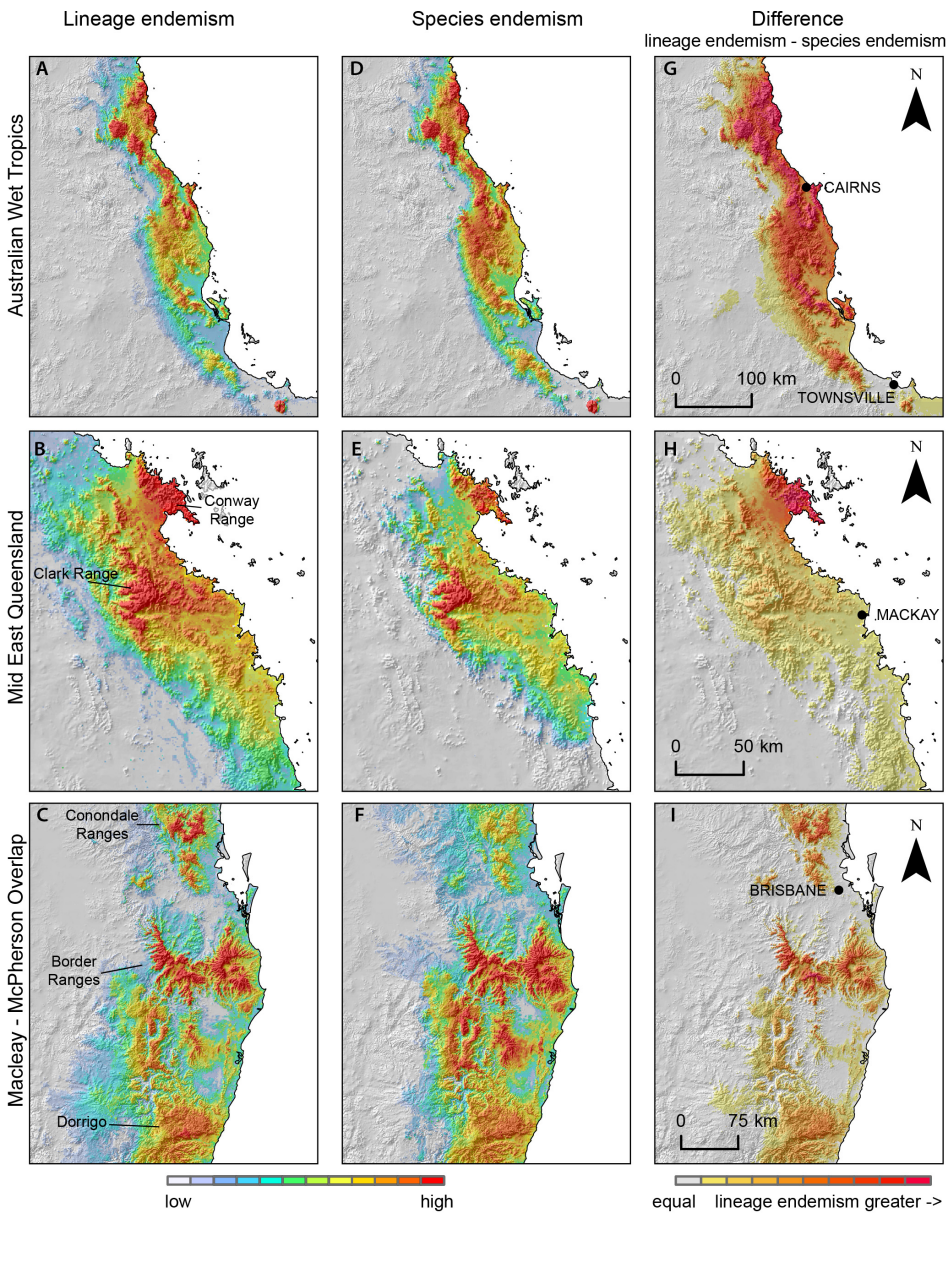
Lineage endemism (left column), species endemism (middle column), and the difference between them (right column), for three areas. Places labelled on panels A-C refer to areas of high endemism. Colours by quantile classes, separately for each panel.

The concentrations of lineage endemism (Fig 4B) highlight a subset of areas with spatially restricted lineage diversity. While diversity and endemism peak in the AWT, there are substantial concentrations of lineage endemism in each region (Fig 5A–5C). Lineage richness (Fig 4C) peaks in the AWT, where overall diversity of rainforest herpetofauna is greatest. Centers of endemism at a fine scale include McIlwraith Range and Cape Melville on Cape York Peninsula, topographically complex areas of the northern and central AWT (Fig 5A), Clark and the Conway Ranges in mid-east Queensland (Fig 5B), the Conondale Ranges area in SEQ and the Border Ranges and Dorrigo area in CEC (Fig 5C). Across the 53 rainforest restricted taxa in this study, the comparison between species and lineage-level endemism highlights substantial differences, particularly at a fine-scale, with areas (red in Figs 4D and 5G–5I) such as the McIlwraith Range, AWT, Conway Range, Conondale Ranges, Border Ranges and Dorrigo area which have substantially more endemic lineage diversity than is apparent at species level, at least for this sample of species. Because of limited data in our study for species south of Sydney, we decided to exclude the endemism results for the SEA region from the subsequent analyses.

### Paleo-stability and other environmental correlates of endemism

With the area of each rainforest region included as a covariate, the GLM models found dynamic stability to be the strongest predictor of lineage endemism. The four best supported predictors together explained 64% of variance in lineage endemism. The standardized model parameters averaged by model weight indicate effect size as follows: dynamic stability (0.37), current rainforest SDM (0.31), topographic roughness (0.18) and annual precipitation (0.09). Models including paleo-stability (dynamic or static) were strongly favoured over those including present environment only (ΔAIC > = 268). Details of the best 5 models are given in S3 Table. Dynamic stability was also the strongest predictor of lineage endemism in the spatial autoregressive model indicating that this result was robust to spatial autocorrelation. Although stability was predicted beyond the boundaries of pre-clearing rainforest, the models of lineage endemism included only current rainforest areas.

**Fig 6:**
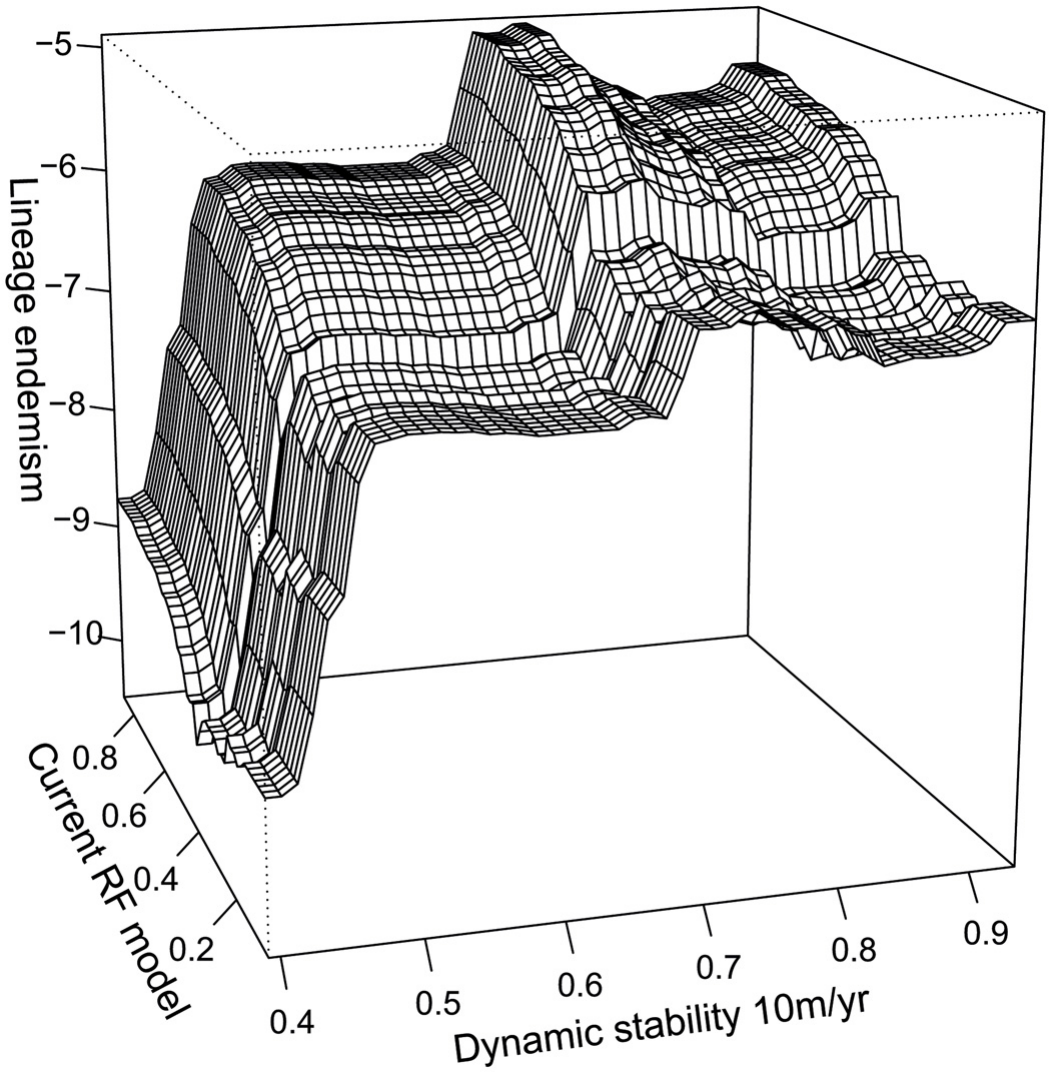
Influence of current rainforest and historical rainforest stability on lineage endemism as predicted by the boosted regression tree model. Values of two predictors are shown: current rainforest habitat suitability, and dynamic stability of that habitat. Other predictors are held to their means. Lineage endemism increases with current rainforest habitat suitability and dynamic stability, but endemism is low in historically unstable areas (dynamic stability < 0.45) regardless of their current habitat.

The boosted regression tree model for all rainforest had a correlation mean of 0.949. The predictors, in order of relative importance were current rainforest SDM (28.7%), dynamic stability (20.9%), mean annual temperature (15.5%), annual precipitation (13.0%), roughness (9.8%), static stability (6.2%) and dry quarter precipitation (5.9%). The boosted regression tree models showed that while dynamic and static stability explains a substantial component of lineage endemism beyond the aspects of current environment tested in these models, this effect was strongest at the low end of the stability spectrum. In fact, endemism was low in the least stable areas [10] regardless of current suitability for rainforest (Fig 6).

Archived data: all the lineage distribution models and results for stability, endemism and richness are archived at DataDryad.org (doi:10.5061/dryad.2pn02).

## Discussion

Measures of both species and lineage endemism identify regions for which a large proportion of diversity is geographically restricted. Species endemism, however, tends to reflect deeper evolutionary and biogeographic processes, but underestimate more recent phylogeographic dynamics. Specifically, within a species, not all lineages are equal in geographic extent, some being highly isolated and restricted while others relatively widespread. Accordingly, regions with high lineage endemism, relative to species endemism, identify hotspots of highly restricted, isolated and independently evolving populations. Identifying such populations and areas is central to comparative phylogeography and could contribute significantly to conservation prioritisation. To date, identifying such areas has largely been qualitative, assessing broad concordance in phylogeographic structure across multiple species, but the approach used here provides a more objective and repeatable approach to spatial analyses of intraspecific diversity.

Within the broad arc of rainforest areas in eastern Australia, we have identified relatively localized centers of endemism. Centers of reptile [69] and frog [67] species endemism in Australia are known at a coarse resolution (50 to 100 km cells), but by working at a finer spatial and taxonomic resolution, we can be far more specific about the particular areas that have retained a disproportionate amount of locally restricted evolutionary diversity. These areas can be considered candidate evolutionary refugia, subject to confirmation across a broader range of taxa.

The lineage endemism analysis presented here includes phylogeographic data for more than half of Australia’s known rainforest-specialist frog and lizard species. In partitioning currently recognized species into their component genetic lineages, we have focused on the most deeply divergent clades in mtDNA phylogeographies. For many of these, especially in the AWT, we have evidence from nuclear genes [12,25,70] and from genetic analyses of hybrid zones [71–73] that such deep phylogeographic clades do indeed correspond with long-isolated and independently evolving evolutionary lineages, which themselves warrant recognition as cryptic species.

### Lineage modelling technique

Phylogeographic data, consisting of locations where genetic data has been obtained for individuals, is inevitably sparse relative to all known distribution points for a given species. Our novel lineage modelling technique seeks to make best use of all available data by integrating gridded environmental data with species occurrence locations, as well as locations of each intraspecific lineage from sequenced specimens. It is likely to be particularly useful in cases where lineages are known from genetic sampling, but the sampling is too sparse to fully characterize their distribution without modelling. Our approach has several advantages. Firstly, it avoids placing sharp boundaries between lineages (as occurs where polygons are drawn around occurrences of a lineage to define its extent of occurrence), except where there is specific evidence of the location of a transition. The inferences drawn using the method are always dependent on the quality of the underlying sampling, but where sampling across a lineage boundary is poor, this is reflected in a gradient of probabilities between two or more lineages (Fig 3C), bringing realistic uncertainty into estimation of the distribution of phylogenetic lineages. Secondly, the method reflects the importance of both geographic distance and barriers of unsuitable habitat as determinants of the location of lineage boundaries, recognizing that the habitats most likely to promote isolation between lineages are particular to each species. We note the broad similarity of our method to that of Tarroso *et al*. [41] which tackles the same problem, but our approach also accounts for the effect of barriers specific to the niche of individual species in estimating lineage ranges. Thirdly, our method avoids ascribing a distinct ecological niche to each lineage, which may often be an inaccurate assumption [48]. Modelling lineages with an SDM would implicitly treat each lineage range as an indication of its niche, when in fact the boundary between adjacent lineages may not indicate any niche differences at all [74]. Our approach assumes that while the overall species distribution is limited by its environmental niche, the intraspecific lineages share the niche requirements of their species, with lineage distributions within that niche defined by isolation and competition. While this likely understates the degree to which lineages have evolved divergent physiological traits or habitat requirements within the species [70,75], we can envisage a future approach that uses available evidence to place each lineage on a continuum from the species niche (as in this study) to an independent niche (equivalent to an SDM).

### Paleo-stability

The dynamic stability analysis identified distinct areas of historically stable rainforest habitat (Fig 4A and S1 Fig) that are broadly consistent with the major areas of pre-clearing rainforest, but show a gradient within them in the degree of historical stability. The static stability results (S1 Fig) showed stable areas in similar locations, but they were smaller and more isolated, particularly in the SEQ region, compared to the dynamic result. The discrepancy in the dynamic result between the moderately low stability in our prediction for the northern AWT and the high stability predicted in previous studies requires further investigation, as does our predicted lack of stability for CYP. One possible explanation lies with the paleo-climate surfaces used for our approach. We used downscaled global circulation models (GCMs). These climate layers differ significantly from those used in previous studies of the AWT, which derived paleo-climate without a GCM by applying adjustments to current climate surfaces based on paleo-ecological estimates of past climate [76,77], or for broader regions using a combination of paleo-ecological evidence and a latitude-based heuristic [21,22]. Despite inherent limitations, the GCM approach enables the extension of paleo distribution and stability analysis to a far larger spatial and temporal extent. A recent assessment by DiNezio & Tierney [58] found that the HadCM3 model we used best captured the Asian-Australian monsoon climate at the LGM overall, but it did not agree with the empirical surrogate for LGM precipitation in the AWT. The remarkably low stability predicted for the CYP region suggests either that the GCM did not capture climate there well, or perhaps that a dynamic model of stability on Cape York would require inclusion of New Guinea rainforest to the north with which it has significant compositional affinity and a long history of fluctuating connectivity [78]. We also note the importance of areas currently offshore in maintaining continuity of rainforest habitat at times of lower sea level when, for some regions, the majority of predicted suitable habitat was beyond the current coastline (S2 Fig).

### Stability and endemism

Paleo-stability as estimated by the dynamic refugia model was a strong predictor of lineage endemism, showing the effect of habitat continuity in supporting the persistence of distinct evolutionary lineages. To avoid attributing current patterns to past processes if there might be a more proximate explanation in current conditions, our correlative models asked whether the observed stability-endemism relationship in frogs and lizards might simply result from endemic rainforest specialists being concentrated in good current rainforest habitat. Our results clearly rejected that proposition, and we concluded that paleo-stability of rainforest has a strong additional role in predicting lineage endemism, and that dynamic stability in particular is clearly supported over static stability. The concept of climate change velocity [79] is often used as an indicator of opportunity for species to persist by dispersing to nearby areas of suitable habitat as climate changes. It is correlated to endemism [7,80]. By simulating dispersal through suitable habitat with a distance constraint, the shifting refugia model also incorporates the same interaction of climate and topography reflected in the velocity index.

The boosted regression tree model suggests that the relationship between stability and endemism is not so much a broad correlation, as a threshold effect whereby the presence of endemic lineages is dependent on a minimum level of dynamic stability. With a few exceptions, lineage endemism was extremely low in historically unstable areas (Fig 6) even where current conditions were highly favourable for rainforest. This is consistent with a large difference in lineage endemism between stable areas with continuous occurrence of lineages, and unstable areas where the currently occurring lineages represent more recent range expansions from refugia [10].

Areas of lesser stability between stable regions align with compositional breaks found in our lineage cluster analysis (Fig 1B) suggesting that historically low dynamic stability may play a role in generating patterns of lineage endemism. For example, two stable areas, in the Border Ranges and the Conondale Ranges area lying just south and north of Brisbane respectively, each display high lineage endemism (Fig 5F), while the gap between them, which spans the city of Brisbane, coincides with a deep break in lineage composition (between SEQ and CEC; Fig 1B and 1C). Similarly, a region of lower stability between Sydney and Newcastle (Fig 4A) separates more stable areas to the north and south by over 100km, and broadly aligns with the southern limit of the Central East Coast region. Are these concordant patterns of instability and lineage turnover due to a causal relationship? It is possible that these unstable areas represent barriers to dispersal that have allowed distinct lineages (or species) to evolve in allopatry. Our cluster analysis result—of islands of stable habitat, which have accumulated distinct assemblages of lineages across multiple taxa—suggests that these barriers might have played an important role in generating endemism, if they persisted over evolutionary time periods sufficient for lineage diversification.

The preponderance of montane areas in the centers of endemism is consistent with our understanding of the effects of late Pleistocene climate cycling. Given that current temperatures are at a warm point relative to recent climate cycles, we would expect that the species and lineages that have their ranges most reduced would be those dependent on cooler temperatures, and perhaps also on the mists which deliver a large proportion of dry-season precipitation in montane coastal rainforests [81]. This range-reduction effect would likely be increased with upslope range shifts [82] under current climate change [83].

### Conservation implications

The implication of this study for conservation is that rainforest areas are not equal in current levels of diversity, nor in their ability to contribute to future diversity. Even within individual rainforest regions, there is wide spatial variation in the amount of locally endemic diversity. The areas of high lineage endemism identified in Figs 4B and 5A–5C support endemic diversity far exceeding surrounding areas within the same region. The comparison of lineage and species endemism highlights places whose importance for conservation may be missed by an analysis at species level (red in Figs 4D and 5G–5I). There is, however, an interesting taxonomic limitation to this comparison. Narrowly distributed lineages result in areas of endemism that may not be apparent at species level, but if an identified lineage is formally described as a species [29,84], its contribution to species endemism and to lineage endemism is the same. Therefore the differences between lineage and species endemism are an indication of both the biological reality, and the state of taxonomy. Obviously, taxonomy affects all analyses of diversity at species level, but here the effect is evident because we have used a different evolutionary unit, defined by phylogeny, and independent of taxonomic attention.

It will be useful to extend this approach to other groups of species to determine how general the patterns of endemism (and thus conservation importance) are to taxa that are less dispersal limited than the rainforest lizards and frogs included in this study. For species with greater dispersal ability, for example some bird taxa and butterfly taxa, one could expect that current habitat suitability would be far more important than local habitat history in defining areas of endemism. Given more reliable estimates of paleo-climate, and even finer-scale lineage endemism data, it may prove possible to test the importance of fine-scale topography and habitat features (e.g. boulder habitats for mesic taxa; [70,85]) by including appropriate layers for topographic buffering of climate [86] and primary productivity in analyses of paleoclimatic stability.

## Conclusions

Our technique for estimating the distribution of intraspecific lineages is likely to be applicable to a range of questions about diversity below species level, including for targeting future data collection to areas with low confidence in lineage assignment. We have identified areas of localized lineage endemism for rainforest herpetofauna, and shown that these areas are strongly related to the continuity of shifting rainforest habitat since the last interglacial (120 ka). In particular, this study supports the dynamic stability model, whereby suitable areas for rainforest could maintain continuity while moving, over time, to track their climatic niche, with some of that continuity dependent on areas of the continental shelf beyond the current coastline. This suggests that chains of suitable habitat, connected through time and space have functioned as a pathway over which older diversity has travelled, to persist into the present.

## Supporting Information

S1 Fig Stability of the climatic niche of rainforest since 120kya. (A) Pre-clearing rainforest from National Vegetation Information System. Stability of rainforest habitat under (B) Static and (C) dynamic 10m / yr models. Blue areas were predicted to have the most continuously suitable rainforest habitat.
(PDF)

S2 Fig Distribution model for rainforest at 3 time periods. Note the large extent of rainforest in areas of current sea, which may have been important in maintaining continuity of rainforest habitat. (A) present model based on the pre-clearing extent of rainforest in S1 Fig (A). (B) projected to 21ka to represent a prediction for the last glacial maximum and (C) projected to 88ka to illustrate an earlier time of large estimated off-shore extent.
(PDF)

S1 Table Correlations between predictors used in the rainforest distribution model. Correlations (Pearson's r) are shown, between environmental predictor layers for those pixels within the 200km buffer used to model rainforest.
(PDF)

S2 Table Variable contributions to the distribution model for present day rainforest (PDF)

S3 Table GLM Model Results. GLM models were fitted with all combinations of up to 5 predictors, with region area included in all models. The top 5 models of 105 models, based on AIC are shown, with their standardized parameter values and Akaike weight. The top model has by far the greatest support, with a weight of 0.97.
(PDF)

S1 Data Location of each specimen with an identified lineage. Count of specimens with an identified lineage, by species
(XLSX)
