# Correction: Lineage Range Estimation Method Reveals Fine-Scale Endemism Linked to Pleistocene Stability in Australian Rainforest Herpetofauna

**DOI:** 10.1371/journal.pone.0169726

**Published:** 2017-01-03

**Authors:** Dan F. Rosauer, Renee A. Catullo, Jeremy VanDerWal, Adnan Moussalli, Conrad J. Hoskin, Craig Moritz

Dr. Conrad J. Hoskin should be included in the author byline instead of the Acknowledgments. He should be listed as the fifth author, and his affiliation is: Zoology & Ecology, College of Science & Engineering, James Cook University, Townsville, Queensland 4811, Australia. The contributions of this author are as follows: conceived and designed the experiments; and contributed reagents/materials/analysis tools.

The correct citation is: Rosauer DF, Catullo RA, VanDerWal J, Moussalli A, Hoskin CJ, Moritz C (2015) Lineage Range Estimation Method Reveals Fine-Scale Endemism Linked to Pleistocene Stability in Australian Rainforest Herpetofauna. PLoS ONE 10(5): e0126274. doi:10.1371/journal.pone.0126274
