# Supplementary material for: Lineage Range Estimation Method Reveals Fine-Scale Endemism Linked to Pleistocene Stability in Australian Rainforest Herpetofauna
Source: PLoS One. 2015 May 28;10(5):e0126274. doi: 10.1371/journal.pone.0126274 (PMC4447262; doi:10.1371/journal.pone.0126274)
Supplement: S1 Fig — (A) Pre-clearing rainforest from National Vegetation Information System. Stability of rainforest habitat under (B) Static and (C) dynamic 10m / yr models. Blue areas were predicted to have the most continuously suitable rainforest habitat. (PDF) [file pone.0126274.s001.pdf]

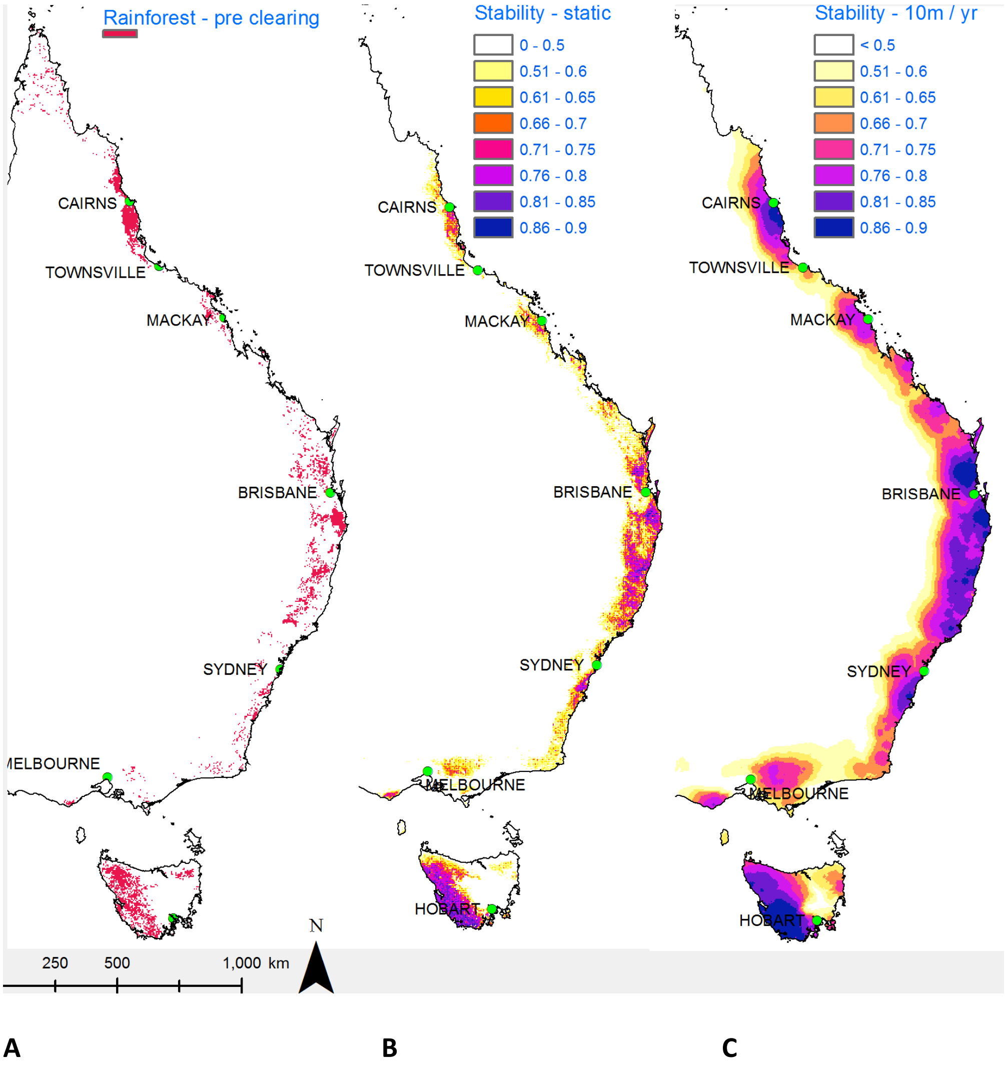

Figure S1. Stability of the climatic niche of rainforest since 120kya. (A) Pre-clearing rainforest from National Vegetation Information System. Stability of rainforest habitat under (B) Static and (C) dynamic 10m / yr models. Blue areas were predicted to have the most continuously suitable rainforest habitat.
