# Supplementary material for: Lineage Range Estimation Method Reveals Fine-Scale Endemism Linked to Pleistocene Stability in Australian Rainforest Herpetofauna
Source: PLoS One. 2015 May 28;10(5):e0126274. doi: 10.1371/journal.pone.0126274 (PMC4447262; doi:10.1371/journal.pone.0126274)
Supplement: S2 Fig — Note the large extent of rainforest in areas of current sea, which may have been important in maintaining continuity of rainforest habitat. (A) present model based on the pre-clearing extent of rainforest in S1 Fig (A). (B) projected to 21ka to represent a prediction for the last glacial maximum and (C) projected to 88ka to illustrate an earlier time of large estimated off-shore extent. (PDF) [file pone.0126274.s002.pdf]

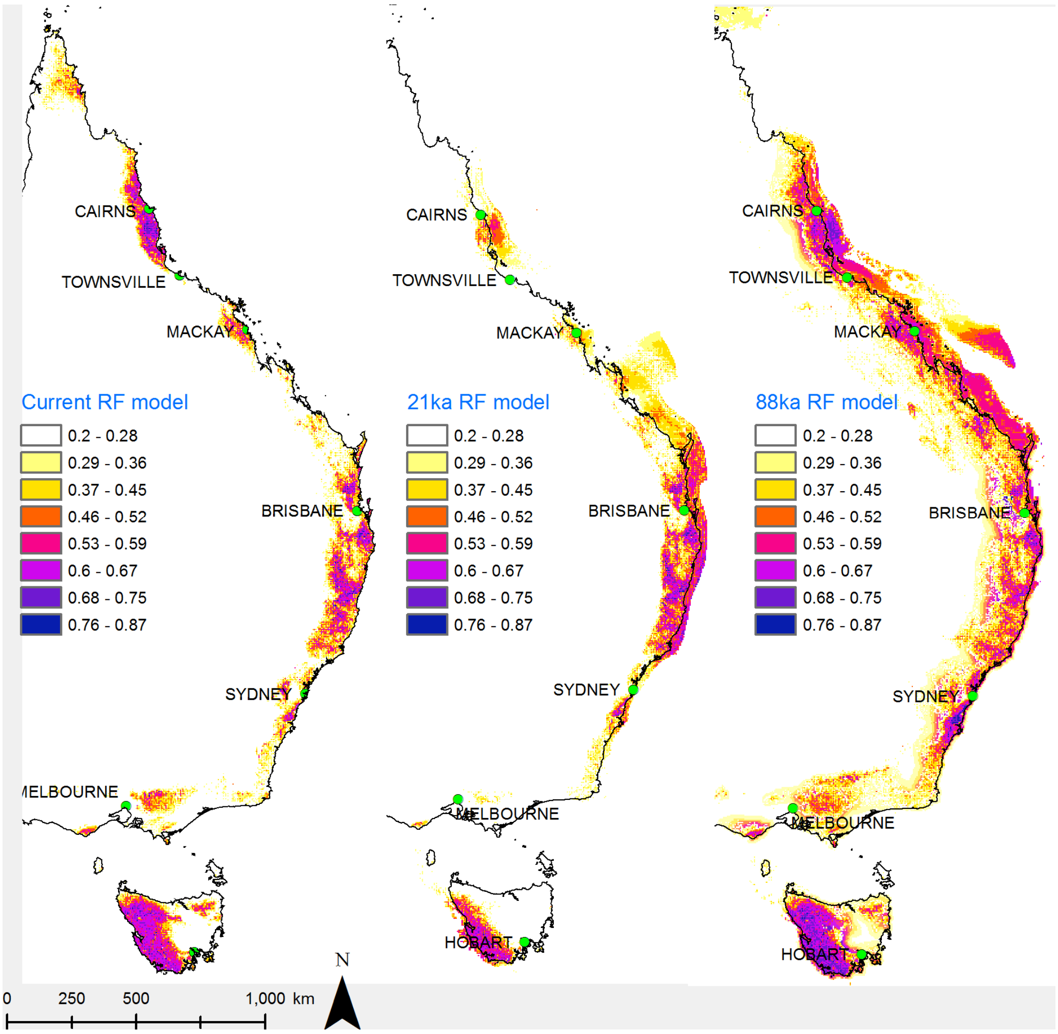

A **B** **C**

Figure S2. Distribution model for rainforest at 3 time periods. Note the large extent of rainforest in areas of current sea, which may have been important in maintaining continuity of rainforest habitat. (A) present model based on the pre-clearing extent of rainforest in Fig. S1(A). (B) projected to 21ka to represent a prediction for the last glacial maximum and (C) projected to 88ka to illustrate an earlier time of large estimated off-shore extent.
