# Supplementary material for: Lineage Range Estimation Method Reveals Fine-Scale Endemism Linked to Pleistocene Stability in Australian Rainforest Herpetofauna
Source: PLoS One. 2015 May 28;10(5):e0126274. doi: 10.1371/journal.pone.0126274 (PMC4447262; doi:10.1371/journal.pone.0126274)
Supplement: S1 Table — Correlations (Pearson's r) are shown, between environmental predictor layers for those pixels within the 200km buffer used to model rainforest. (PDF) [file pone.0126274.s003.pdf]

Table S1: Correlations between predictors used in the rainforest distribution model

Correlations (Pearson's r) are shown, between environmental predictor layers for those pixels within the 200km buffer used to model rainforest.

	bioclim 04	bioclim 10	bioclim 11	bioclim 12	bioclim 15	bioclim 16	bioclim 17	elevation range	slope
**bioclim 01**	-0.489	0.963	0.975	0.096	0.878	0.583	-0.831	-0.422	-0.471
**bioclim 04**		-0.240	-0.670	-0.610	-0.683	-0.791	0.341	0.084	0.094
**bioclim 10**			0.882	-0.078	0.766	0.411	-0.823	-0.450	-0.502
**bioclim 11**				0.236	0.919	0.699	-0.795	-0.384	-0.428
**bioclim 12**					0.277	0.816	0.288	0.278	0.281
**bioclim 15**						0.743	-0.791	-0.254	-0.288
**bioclim 16**							-0.305	0.008	-0.011
**bioclim 17**								0.452	0.491
**elevation range**									0.910
