# Supplementary material for: Lineage Range Estimation Method Reveals Fine-Scale Endemism Linked to Pleistocene Stability in Australian Rainforest Herpetofauna
Source: PLoS One. 2015 May 28;10(5):e0126274. doi: 10.1371/journal.pone.0126274 (PMC4447262; doi:10.1371/journal.pone.0126274)
Supplement: S2 Table — (PDF) [file pone.0126274.s004.pdf]

Table S2: Variable contributions to the distribution model for present day rainforest

Percent contribution	Variable	Description
**56.3**	bioclim 12	Annual precipitation
**13**	bioclim 04	Temperature seasonality
**11.7**	slope	Slope
**11.4**	bioclim 17	Precipitation of driest quarter
**2.7**	bioclim 15	Precipitation seasonality
**2.2**	elevation range	Elevation range
**1**	bioclim 11	Mean temperature of wettest quarter
**0.6**	bioclim 01	Annual mean temperature
**0.6**	bioclim 10	Mean temperature of warmest quarter
**0.3**	bioclim 16	Precipitation of wettest quarter
