# Supplementary material for: Lineage Range Estimation Method Reveals Fine-Scale Endemism Linked to Pleistocene Stability in Australian Rainforest Herpetofauna
Source: PLoS One. 2015 May 28;10(5):e0126274. doi: 10.1371/journal.pone.0126274 (PMC4447262; doi:10.1371/journal.pone.0126274)
Supplement: S3 Table — GLM models were fitted with all combinations of up to 5 predictors, with region area included in all models. The top 5 models of 105 models, based on AIC are shown, with their standardized parameter values and Akaike weight. The top model has by far the greatest support, with a weight of 0.97. (PDF) [file pone.0126274.s005.pdf]

**Table S3: GLM Model Results**

GLM models were fitted with all combinations of up to 5 predictors, with region area included in all models. The top 5 models of 105 models, based on AIC are shown, with their standardized parameter values and Akaike weight. The top model has by far the greatest support, with a weight of 0.97.

| (Intercept) | Dynamic stability | Static stability | Mean annual temperature | Mean annual precipitation | Driest quarter precipitation | Current rainforest SDM | Topographic roughness | Region area | Delta AIC | Weight |
|---|---|---|---|---|---|---|---|---|---|---|
| 0 | 0.3650 | - | - | 0.0895 | - | 0.3054 | 0.1848 | -0.5421 | 0 | 0.97333 |
| 0 | 0.4302 | -0.1093 | - | - | - | 0.4064 | 0.1707 | -0.5654 | 7.572 | 0.02208 |
| 0 | 0.4267 | - | - | - | -0.0718 | 0.3613 | 0.1564 | -0.5638 | 10.846 | 0.00429 |
| 0 | 0.3931 | - | 0.0506 | - | - | 0.3420 | 0.1809 | -0.5494 | 16.572 | 0.00024 |
| 0 | 0.3911 | - | - | - | - | 0.3464 | 0.1667 | -0.5852 | 19.908 | 4.63E-05 |
